# Kinetic pathway of HIV-1 TAR cotranscriptional folding

**DOI:** 10.1093/nar/gkae362

**Published:** 2024-05-13

**Authors:** Lei Jin, Sicheng Zhang, Zhenwei Song, Xiao Heng, Shi-Jie Chen

**Affiliations:** Department of Physics and Institute of Data Science and Informatics, University of Missouri, Columbia, MO 65211, USA; Department of Physics and Institute of Data Science and Informatics, University of Missouri, Columbia, MO 65211, USA; Department of Biochemistry, University of Missouri, Columbia, MO 65211, USA; Department of Biochemistry, University of Missouri, Columbia, MO 65211, USA; Department of Physics and Institute of Data Science and Informatics, University of Missouri, Columbia, MO 65211, USA; Department of Biochemistry, University of Missouri, Columbia, MO 65211, USA

## Abstract

The Trans-Activator Receptor (TAR) RNA, located at the 5′-end untranslated region (5′ UTR) of the human immunodeficiency virus type 1 (HIV-1), is pivotal in the virus’s life cycle. As the initial functional domain, it folds during the transcription of viral mRNA. Although TAR’s role in recruiting the Tat protein for trans-activation is established, the detailed kinetic mechanisms at play during early transcription, especially at points of temporary transcriptional pausing, remain elusive. Moreover, the precise physical processes of transcriptional pause and subsequent escape are not fully elucidated. This study focuses on the folding kinetics of TAR and the biological implications by integrating computer simulations of RNA folding during transcription with nuclear magnetic resonance (NMR) spectroscopy data. The findings reveal insights into the folding mechanism of a non-native intermediate that triggers transcriptional pause, along with different folding pathways leading to transcriptional pause and readthrough. The profiling of the cotranscriptional folding pathway and identification of kinetic structural intermediates reveal a novel mechanism for viral transcriptional regulation, which could pave the way for new antiviral drug designs targeting kinetic cotranscriptional folding pathways in viral RNAs.

## Graphical Abstract

**Graphical Abstract:**
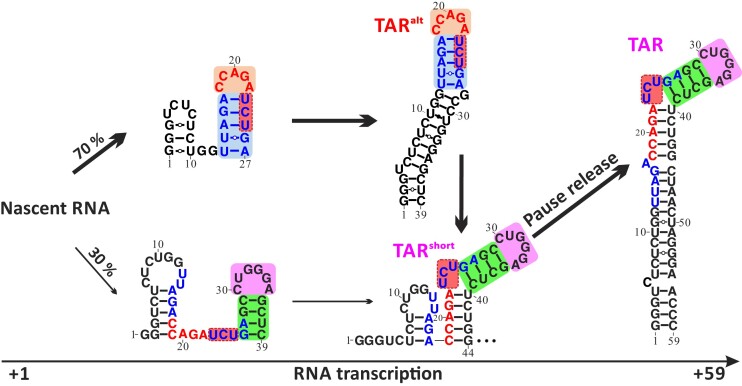

## Introduction

**Figure 1.**
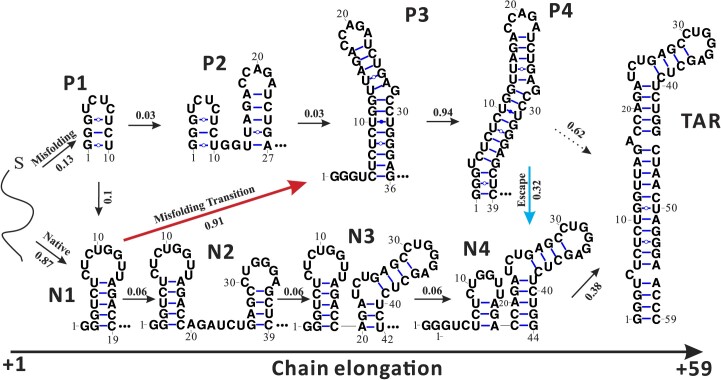
The cotranscriptional folding pathway of HIV-1 TAR (1–59 nt): the pause-pathway P1–P4 and the native-pathway N1–N4. Inter-pathway transitions are shown in red arrow for N1 to P3 and the blue arrow for P4 to N4. The fractional population flux for each transition is shown on the arrow. See Supplementary Figures S1 and S2 for the detailed transition pathways from P4 to TAR and N4 to TAR. The population fluxes into and out of a structure represent the respective fractional population flows, corresponding to the population partitioning among the different transitions. For a given structure, the total in-flux is equal to the total out-flux, and for a given chain length, the sum of the in-flux over all the structures is equal to 1.

The 5′ untranslated region (UTR) of HIV-1 spans approximately 360 nucleotides and encompasses crucial functional domains. These domains comprise the transactivating response region (TAR), Poly-A, primer binding site (PBS), dimerization initiation site (DIS), splicing donor (SD), and Psi. Each of these domains serves an indispensable role in viral replication (1–3), with their functionality intricately linked to structures and dynamic alterations. In particular, the folding of TAR into a hairpin structure that includes a -UCU- bulge loop (Figure 1, the structure on the right) plays a crucial role in the binding of the Trans-Activator of Transcription (Tat) protein (4,5). The interaction between Tat and TAR subsequently recruits cyclin-dependent kinase 9, leading to the phosphorylation of the C-terminal domain of the RNA polymerase II (RNAP II) subunit (6–9). This process facilitates the transition to the highly efficient elongation phase of transcription, and thus trans-activates HIV-1 transcription (4,6,10–16).

In general, promoter-proximal pausing plays, a vital role during the transcription process of protein-encoding genes, observed in mammals and *Drosophila* (17–19). Recent progress in sequencing technologies has facilitated extensive investigations into the mechanisms of Pol II pausing, unveiling its indispensable role as a regulatory checkpoint during transcription (20). Furthermore, Pol II engages in dynamic interactions with various host proteins that inhabit and promote differential gene expression required for development in model organism and mammals (21–25).

In the case of HIV-1, the promoter-proximal pausing is coordinated by the nucleic acid sequence. Notably, a significant transcriptional pause occurs at +62 nt, which is induced by Pol II’s reverse translocation (backtracking) on the nascent non-native TAR hairpin structure. The rearrangement of the nascent RNA into TAR subsequently enables escape from the pause through forward tracking (26). While extensive research has proposed the involvement of promoter-proximal pausing in Tat-mediated regulation of HIV-1 transcription, polyadenylation, and splicing (26–31), the detailed cotranscriptional folding pathway of TAR, especially the process by which the non-native TAR hairpin (kinetic intermediate) prompts Pol II to backtrack for pause and structure rearrangement to the native TAR for pause escape, remains uncharacterized.

In this study, we investigate the structural and kinetic roles of TAR in transcriptional pausing and de-pausing. An RNA folds as it’s transcribed. During this process, the transcription rate often exceeds the structural refolding rate of the nascent chain, resulting in a kinetic (non-equilibrium) control of RNA structure formation (32–36). Recent studies have demonstrated that RNA nascent structures play a pivotal role in viral gene expression and regulation (11,37–40). However, our current understanding of the structures and folding pathways and their contributions to RNA functionalities remains limited (41). We focus on the cotranscriptional folding pathway and structural conversion of TAR in HIV-1. Experimental determination of the cotranscriptional RNA folding poses challenges due to the presence of lengthy RNA chains, resulting in an intractably large conformational space (42) to be explored. To address this issue, various theoretical and computational approaches have been developed (43–48). In this study, we employ our recently developed (2D structure-based) energy landscape zooming (LZ) model for predicting cotranscriptional folding (47). Unlike previous approaches that exclude pseudoknots (47), our current model incorporates the essential pseudoknots into the conformational ensemble. The model delineates two parallel pathways that play a significant role in the transition from the (pause-inducing) non-native intermediate (P4 RNA) to the native TAR conformation. The secondary structure of the kinetic TAR structure is supported by NMR, and the data is in accordance with the presence of the non-native TAR structure at the pausing site (+62 nt) and the predicted kinetic pathway. Furthermore, the predicted pause duration is quantitatively consistent with the experimental observation (26). Additionally, our model predicts a non-native TAR with an altered -UCU- bulge loop, which further agrees with the enzymatic probing of alternative TAR structures (49). For the HIV-1 TAR system, in a previous study (47), because the original LZ model did not treat the transcription pause and pseudoknot-assisted transitions, we investigated only the upstream sequence from the pause site. The P4 and N4 structures were predicted in the previous study, however, how they evolve to the final 59-nt native TAR was not investigated, and the kinetic mechanisms for triggering and escaping pauses, and the biological implications of the kinetic mechanisms across different HIV variants were not studied.

Our computational and experimental findings reported in this work highlight the importance of short-lived transient structures in the conformational transformation of TAR and the subsequent process of pause-escaping. Given that the cotranscriptional folding and the transcriptional pauses of TAR can impact the folding pathway of the downstream sequence, this study holds significant implications for our understanding of HIV-1 gene expression. Moreover, these findings can offer structural and kinetic insights into novel antiviral strategies targeting cotranscriptionally folded nascent RNA structures.

## Materials and methods

### Predicting RNA folding kinetics

We employ the energy landscape zooming model, a recently developed approach (47), to predict RNA cotranscriptional folding kinetics. The key ingredient of the LZ method is the coarse-graining of the conformational space by utilizing discrete low-free energy (stable/metastable) structures and dividing the conformational space into partitions centered around the stable structures. Additionally, we use the Vfold model, which can handle pseudoknots and non-canonical base pairs, for the computation of free energy (50–52). Specifically, our computation consists of several steps, which are outlined below (further details can be found in the Supporting Information, SI):

- Identify and select all stable/metastable (low-free energy) helices.
- Construct structural partitions by assembling the stable and metastable helices.
- Compute transition rates between different partitions through kinetic Monte Carlo simulations (KMC) (47).
- Integrate the transition rates into the master equation to compute the kinetics and pathways in the partition-based transition network.

The LZ model utilizes Vfold2D (52), a 2D RNA structure folding model, for RNA structure generation and free energy evaluation. LZ employs KMC to calculate transition rates between different states and computes the population kinetics by solving the Master Equations. We note that KMC simulation has been applied to model RNA folding kinetics, tested for RNA hairpin folding and structural rearrangements, including those involving pseudoknots, and validated for the scaling of the simulated time to match the experimental time (65).

### HIV-1 5′UTR cotranscriptional folding

We model mRNA elongation as a stepwise process with consecutive addition of nucleotides at a predefined speed (the transcription elongation speed). Following the addition of each nucleotide, the folding energy landscape is updated to reflect the sequence elongation, thereby allowing the newly extended nascent RNA chain to fold on the updated energy landscape. In this study, to be consistent with the in vitro experimental conditions (26), we adopt a relatively slow transcription speed of 4 nt/s. Additionally, we also examine the folding with two higher transcription speeds of 20 and 40 nt/s, which correspond to the transactivated transcription speeds of RNA Polymerase II determined in different experiments (53–62). HIV-1 TAR sequence from NL4-3 strain is used in this study.

Using the transition rates between different structures, we calculate the population flux *P*_*ij*_ for the transition from structure *i* to *j*:


(1) \begin{equation*} P_{ij}=\int\limits\left[k_{ij} p_i(t)-k_{ji} p_j(t)\right] dt \end{equation*}

where *k*_*ij*_ and *k*_*ji*_ represent the transition rates from structure *i* to *j* and from *j* to *i*, respectively, and *p*_*i*_(*t*) and *p*_*j*_(*t*) are the populations of structures *i* and *j* at time *t*, respectively. We infer the transition pathway from the population fluxes.

### Pseudoknots and transcriptional pause

Pseudoknots play a crucial role in kinetic transition between the different structures. By forming transient crossing base pairs, pseudoknots effectively decrease the free energy barrier for structural transitions (63–65). The original LZ model employed in the cotranscriptional folding model does not consider pseudoknots in the kinetic pathways (47,65). In this study, we incorporate the formation and disruption of pseudoknots along the kinetic pathways in the LZ model. When a transcriptional pause occurs, the elongation of the RNA chain is temporarily halted while the folding of the RNA continues (54,66,67). To simulate the transcriptional pause, we freeze the chain elongation when the pause-triggering condition is met, e.g. the formation of the P4 structure for the TAR system. During the pause period, the nascent RNA continues to fold (without chain elongation). Chain elongation is resumed when the pause-escaping condition is met, e.g. the native TAR structure is formed for the TAR system.

### RNA synthesis and purification

The P4 RNA was synthesized as previously described (68). Briefly, DNA template 5′-GAG CTC CCA GGC TCA GAT CTG GTC TAA CCA GAG AGA CCC TAT AGT GAG TCG TAT TAA TTT C-3′ was heat annealed with 5′-GAA ATT AAT ACG ACT CAC TAT AG (synthesized at IDT) for T7 polymerase binding. In vitro transcription was carried out in 40 mM Tris–HCl pH 8.0, 10 mM spermidine, 22.5 mM MgCl_2_, 0.01% (v/v) Triton X-100, 5 mM DTT and 12 mM of each NTP. The RNA was purified by denaturing sequencing gel, recovered by elutrap (Whatman), and washed using 2 M NaCl followed by ddH_2_O using Amicon ultra-centrifugal units (3K MWCO). The concentration of RNA was determined by absorbance at 260 nm using Nanodrop (Thermo Scientific).

### NMR spectroscopy

The P4 RNA was prepared at 800 μM in 10% D_2_O + 90% H_2_O buffer containing 10 mM Tris–HCl, pH 5.5 and 1 mM MgCl_2_.The RNA was refolded by heating up to 95^○^C for 3 min and rapidly cooling on ice. One dimensional and two dimensional 1H–1H NOESY were collected at 278 K on a Bruker Avance III 800 MHz spectrometer equipped with TCI cryoprobe (NMR Core, University of Missouri). The NMR data were processed by NMRPipe (69) and analyzed by NMRViewJ (70).

## Results

### Kinetic folding pathway of TAR

In vitro experiments indicate the presence of a non-native intermediate (Figure 1, P4 RNA) at the transcriptional pausing site, spanning from +62 to +68 of the 5′ UTR (26). It is important to note that the non-native intermediate observed at the pausing site is considerably less stable compared to the native TAR structure in its full length, and is an intermediate state in the cotranscriptional folding process of the native TAR structure. The folding of the 59-nt TAR sequence is expected to follow the kinetic folding pathway.

### Read-through transcription

To compare our theoretical modeling results with experimental findings, we conducted a read-through cotranscriptional folding simulation without the pause at +62 U. The transcriptional rate was set at 4 nt/s, which matches recent studies indicating a transcription rate of approximately 0.5 kb/min during the initiation stage of Pol II (58), and a transcriptional rate of around 4 nt/s for the HIV-1 5′ UTR as reported in Palangat *et al.* (26).

As depicted in Figure 1, our cotranscriptional folding simulation reveals two main parallel pathways: the pause-pathway and the native-pathway. The pause-pathway (P1 to P4) involves a 39-nt meta-stable hairpin P4, which has been previously identified as the hairpin responsible for interacting with the Pol II RNA and transcriptional pausing (26). The native-pathway (N1 to N4) leads to the formation of a 44-nt native-like TAR hairpin (N4), featuring a -UCU- bulge loop that serves as the Tat binding site.

Additionally, our simulation revealed three inter-pathway transitions: P1 to N1, N1 to P3 and P4 to N4. Notably, before the elongation reaches nucleotide 42 (see structure N3), marking the initial formation of the Tat-binding -UCU- bulge loop in the native-pathway, the transition from the native-pathway to the pause-pathway is predominant. However, after nucleotide 42 (N3), the inter-pathway transition is reversed, primarily driven by the transition from P4 to N4 which results in the formation of the native TAR structure.

**Figure 2.**
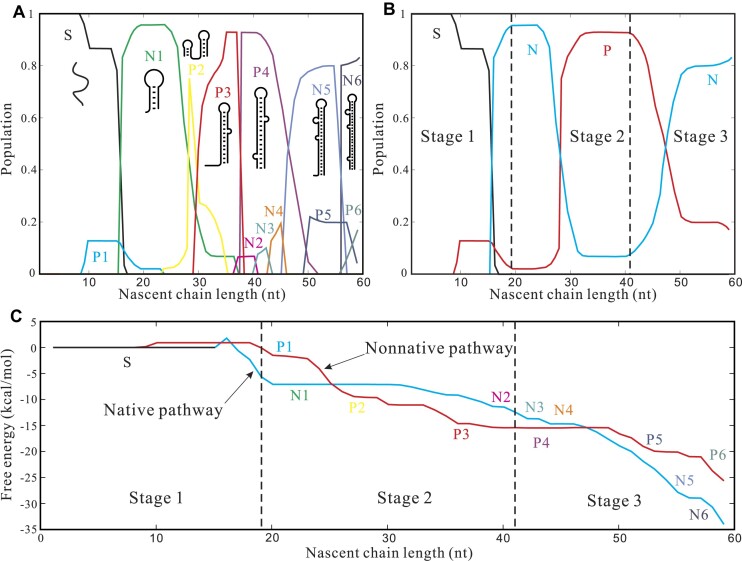
(**A**) The population kinetics during the cotranscriptional folding. Populations are calculated from Eqs. (S2) and (S3) in the SI. Illustrations for the structures with the highest population along the chain elongation are shown. (**B**) The population kinetics of the pause-pathway (P), the native-pathway (N), and the unfolded state (S) during different transcription stages. (**C**) Free energy profiles along the native-pathway and the pause-pathway, respectively. P5, P6, N5 and N6 in (A) and (C) correspond to the 2D structures shown in Supplementary Figures S1 and S2.

When the transcription pause occurs at +62U, about 18 nucleotides from +45C to +62U are protected in the RNAP–DNA complex and do not participate in folding (71). To gain quantitative insights into the detailed structural distribution, we computed the fractional population of different structures at various elongation stages, up to +44G, as shown in Figure 2A. At the elongation length of 44 nucleotides, the populations of the pause hairpin P4 and the native-like TAR hairpin N4 are approximately 75% and 25%, respectively. This predicted population ratio of 75%:25% for P4 and N4 aligns closely with the experimental result (26), which calculated a ∼70% maximum pausing efficiency. The theory-experiment agreement supports the validity of the cotranscriptional folding model.

Furthermore, our calculations reveal a substantial population flux towards the native-like TAR structures N5 and N6 from the non-native intermediate structures P5 and P6 (see Supplementary Figures S1 and S2). When all the 59 nucleotides of the full TAR sequence are released from RNAP, making them available for folding, the model predicts over 80% of the total population to be occupied by the native (or native-like) structures. This result indicates that at low transcription elongation speed of 4 nt/s (26), the native (or native-like) structures dominate the population.

To further investigate the parallel folding pathways, we calculate the population flux within each of the two pathways as


(2) \begin{equation*} {P_{\text{path}}(t)=\sum _{\text{path}} p_i(t)} \end{equation*}

where *p*_*i*_(*t*) represents the population of state *i* at time *t*, and *P*_path_(*t*) denotes the population of all the states on the given pathway.

The population kinetics (i.e., the time-dependence of the population) for each state within the two pathways were calculated using Eq. (S3). The overall populations for both pathways, as calculated from Equation (2), are represented in Figure 2B. The predicted population kinetics shows that the two folding pathways coexist in the early stages of transcription. The entire folding process can be divided into three transcription stages:

- Stage 1 (elongation from 1-nt to 19-nt): During this stage, the cotranscriptional folding primarily follows the native pathway, with a fractional population >0.9.
- Stage 2 (elongation from 20-nt to 42-nt): In this stage, there is a growing population in the pause-pathway, causing it to gradually become the major pathway.
- Stage 3 (elongation from 44-nt to 59-nt): In this stage, due to the inter-pathway transitions from the pause-pathway to the native-pathway, the native-pathway once again becomes dominant. However, the pause-pathway is not entirely eliminated at the end of transcription, and structures from both parallel folding pathways can coexist. The population flux from the pause-pathway to the native-pathway persists, indicating that the population distribution of the system undergoes continuous evolution.

As shown in Figure 2C, prior to the elongation reaching 42-nt, the free energy of the pause hairpin P4 is significantly lower than the free energies of the native-like (partially folded) TAR hairpins N3 and N4. Moreover, the free energies of the native-like (partially folded) TAR hairpins from N3 to N5 decrease as the chain elongates from 42-nt to 50-nt. At 59-nt, the fully folded native TAR hairpin N6 demonstrates a free energy of −30.7 kcal/mol, in contrast to the −22.7 kcal/mol of the non-native hairpin P6. This free energy difference suggests that the native TAR hairpin is the thermodynamically stable structure for the sequence, whereas the pause structure represents a transient metastable state of the nascent chain formed along the cotranscriptional folding pathway.

### Pseudoknot-assisted transitions

As depicted in Figure 1, the inter-pathway transitions play a crucial role in determining the folding pathway of TAR. Specifically, the transitions from P1 to N1, N1 to P3, and P4 to N4 are of significant importance. Understanding these inter-pathway transitions is crucial for designing effective drugs that target the kinetic intermediates formed during cotranscriptional folding. In this study, we focus on the latter two transitions, as they involve important structures in the late stage of the folding pathways.

In the structural rearrangement, the dominant transition trajectory with a low energy barrier involves base pair exchanges: an existing base pair is disrupted, followed by the subsequent formation of a new base pair, resulting in a low energy cost (65). This base pair exchange mechanism often involves the formation of pseudoknots (PKs) as the intermediate states to effectively facilitate structural rearrangements. By incorporating the possible pseudoknots in the structural ensemble, our LZ model enables the prediction of pseudoknot-assisted structural rearrangement and pseudoknot-containing folding pathway.

**Figure 3.**
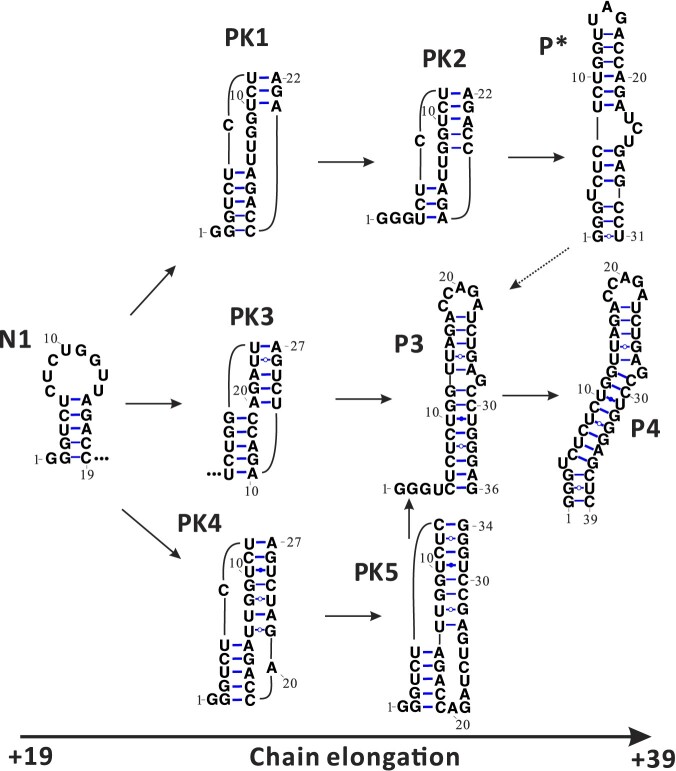
Transition between the native-pathway (N1) and the pause-pathway (P3, P4). Five pseudo- knot intermediates and three parallel pathways are involved in the transition. We note that the predicted formation of P4 and P* is consistent with the experimentally suggested non-native structures in the cotranscriptional folding (26,49).

**Figure 4.**
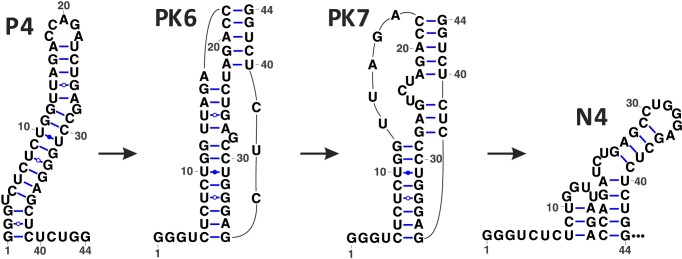
Transition from the non-native intermediate (pause hairpin) P4 to the native-like structure N4. The PK-assisted pathway as the dominant pathway involves two pseudoknot intermediates PK6 and PK7.

Our computational analysis suggests that approximately 90% of the total population undergoes the N1 to P3 transition, as illustrated in Figure 3, through PK-assisted pathways involving the folding and unfolding of transient pseudoknots (PK1–PK5). Instead of completely unfolding N1 and subsequently refolding from an unfolded state, this transition occurs via base pair exchanges described above and the formation of pseudoknotted intermediates during the elongation process from +19C to +39C. As depicted in Figure 3, a metastable hairpin structure, referred to as P*, is formed along the PK-assisted pathway. Notably, a recent experimental study has also proposed the early formation of P* during transcription (49). Furthermore, it is worth mentioning that the transition rate from N1 to P3 is higher compared to that from P* to P3, primarily due to the formation of PK3. Due to the low transition rate from P* to P3, a small population of P* folds directly to the native TAR structure without passing through P3 and P4; see Supplementary Figure S3. Approximately 30% of the P4 population follows the PK-assisted pathway to transition to the native-like TAR structure N4, as shown in Figure 4, with the formation of pseudoknots PK6 and PK7 as kinetic intermediates. Importantly, the pseudoknot-assisted routes exhibit significantly faster transition rates compared to the pseudoknot-free pathways, because the formation of pseudoknot reduces the energy barrier of structural transition, which inversely correlates with the kinetic rate (65). Our calculations showed that the pseudoknot-free transition rate from P4 to N4 is around 4.3 × 10^−2^*s*^−1^ compared to 2.3 × 10^−1^ s^−1^ for the pseudoknot-assisted transition pathway. In the pseudoknot-assisted transition pathway, the PK7 to N4 transition (Figure 4) involves the rearrangement of the 8-bp long helix stem from 5C-G36 to 12G-C29 and thus has a high energy barrier which results in a rate-limiting step.

### Cotranscriptional folding intermediates

**Figure 5.**
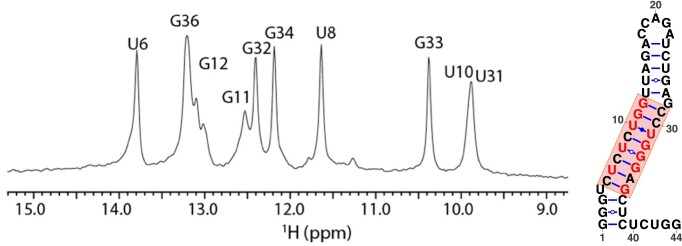
Left: Imino proton spectra for the 39-nt intermediate structure in the cotranscriptional folding. Right: Predicted secondary structure of the 39-nt intermediate structure P4. The NMR assigned nucleotides are highlighted in red. The helix containing the assigned base pairs is outlined in a red box.

To experimentally examine the RNA intermediates formed during cotranscriptional folding, nucleotide 1–39 was synthesized and purified to mimic the nascent RNA outside of the Pol II RNA exit tunnel. 1D and 2D imino proton spectra were collected for the RNA at 278 K, see Figure 5. These imino protons were assigned to residues in the middle stem of P4 (U6–G12 and U31–G34) shown in Figure 1. Interestingly, no imino signals from the top or bottom stem were detected, likely caused by rapid exchange with solvent due to internal motions. This is consistent with the predicted pseudoknot-assisted structure transition from P4 to PK6 (shown in Figure 4) that the internal motions in the top and bottom stems provide flexibility to allow the top loop residues to base pair with nucleotide 40–44 and form intermediate PK6 as transcription proceeds.

### Transcriptional pause

Previous experiments have identified a transcriptional pause at the early stage of HIV-1 transcription (54,66,67). In order to examine the impact of this pause on the cotranscriptional folding pathway of TAR, we incorporated a transcriptional pause into the folding simulation. The transcriptional pause of HIV-1 TAR occurs at nucleotide position +62 (26). During transcription, the 18 nucleotides at the 3′-end of the nascent RNA are protected within RNAP II (26), and the steric impediment from RNAP II prevents these 18 nucleotides from participating in RNA folding. This is based on the observation that 17–19 nucleotides of the RNA’s 3′-end in the exit tunnel of polymerase were protected from exogenous ribonuclease digestion (71). Therefore, as outlined in the Methods, we consider only the first 62 − 18 = 44 nucleotides (from positions +1 to +44) to participate in RNA folding before pause release. Following experimental findings (26), we assume that the non-native intermediate P4 can induce the transcriptional pause (54,66,67). After the 44th nucleotide is transcribed, elongation of the RNA chain is paused while the folding of the 44-nt transcribed chain persists. Upon the formation of the native-like TAR structure N4 with the -UCU- bulge loop, Tat protein can bind to the TAR structure, and the transcription is resumed. Therefore, realistically a reverse transition from the native-like TAR N4 to the non-native intermediate P4 does not occur and is thus not allowed in the kinetic model.

**Figure 6.**
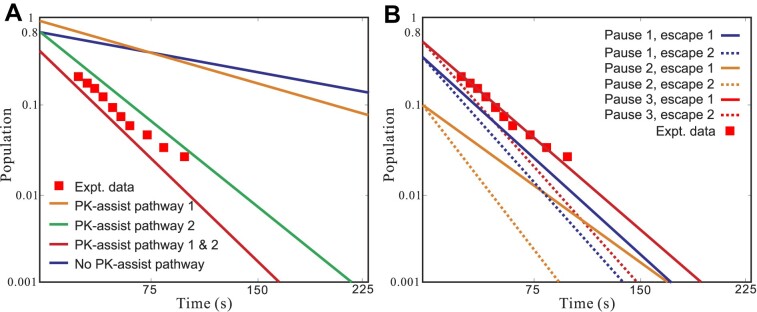
The non-native hairpin P4 converts to native-like structure N4 during the transcriptional pause. (**A**) The time-dependence of the population of the non-native hairpin P4 (solid line) predicted with/without pseudoknot-assisted transition pathway 1 and/or pathway 2 compared with the experimental data (symbol) (26). The transcription pause is stimulated when +62U is transcribed before the pause hairpin P4 is transformed into the native(-like) TAR. The PK-assisted pathways 1 and 2 are shown in Figure 3 and Figure 4, respectively. (**B**) The time-dependence of the population of the transcriptional pause structures (solid and dashed line) predicted with different pause controlling mechanisms. Pause 1: pause stimulated at +62U with P4 formed. Pause 2: pause stimulated at +62U with P* formed. Pause 3: pause stimulated at +62U with P4 or P* formed. Escape 1: pause released with the formation of native TAR. Escape 2: pause released when structure P4 or P* no longer exists. Experimental data for the paused sequence are shown in symbol (26).

To investigate how the pseudoknot (PK)-assisted transition could impact the folding pathway and population kinetics, we performed simulations of the pause with the two PK-assisted pathways (pathways 1 and 2 in Figures 3 and 4, respectively) being technically turned on and off. We then calculated the pausing half-lifetime for each case. As shown in Figure 6A for the theory-experiment comparisons, turning on pathway 2 notably enhances the accuracy of the predicted pausing kinetics. This is evident from the simulation results for turning on pathway 1 while turning off pathway 2 (orange line), turning off pathway 1 and turning on pathway 2 (green line), and turning on both pathways 1 and 2 (red line). As shown in the red line in Figure 6A, the population (probability) of the RNA to remain in the paused state decays over time from its initial value of 0.65. The predicted pause half-time is approximately 15 seconds, which aligns with the calculated pausing half-life of 22 s based on the experimental data (26). These findings indicate the presence of a pause-escaping mechanism facilitated by the structure transition from the pause hairpin (P4) to the native TAR hairpin (N4 and the full, native TAR structure). Additionally, the PK-assisted pathway contributes significantly to the pause duration. As shown in Figure 6A, the inhibition of PK-assisted pathways would result in an increased pause duration time. The impact of the PK-assisted pathway from P4 to N4 is particularly noteworthy, as it leads to a substantial increase in the pause half-time from 15 s to approximately 150 seconds, because the formation of the intermediate PKs can reduce the overall transition free energy barrier for over 6 kcal/mol. This result suggests that the PK-assisted pathway dominates the P4 to N4 transition. Without the PK-assisted pathway, the escape from the pause state would be significantly slowed down.

In the pause and pause-escape mechanisms described above, the pause-inducing hairpin P4 and its transition to the native-like N4 play a central role. However, to explore different possibilities, we have also examined alternative mechanisms by considering different pause-stimulating structures, including P*. Our investigation involved evaluating the relaxation (decay) kinetics of their populations. In particular, we considered structure P* due to its fractional population of approximately 10% as transcription enters the pause site at +62U. As shown in Figure 6B, if the P* structure induces the pause (Pause 3 in Figure 6B), the predicted pause duration is 28 s, which closely corresponds to the experimental result. The finding suggests that the formation of N4 leads to pause-escape (Escape 1 in Figure 6B). We are currently unable to exclude this mechanism because, based on the fundamental mechanism of transcriptional pauses in RNAP II, any upstream hairpin formed in the nascent RNA chain (such as P4 and P* in this case) could be a contributing factor to the transcriptional pause.

### Effect of transcription speed

The cotranscriptional folding pathway is intricately influenced by the competition between several rate processes including inter-pathway transitions that involve the folding and unfolding of nascent structures. These dynamic processes depend on the transcription speed at which the RNA chain elongates. In the cell, the transcription speed (= chain elongation rate) depends on multiple transcription factors as well the gene sequence content (72). To obtain the general cotranscriptional folding pathways of TAR, we used the average transcription speeds extrapolated from the experimental data (26). Generally, the inter-pathway transitions discussed above exhibit a relatively slower rate compared to transcriptional elongation. However, altering the transcription speed can yield a substantially different folding pathway. Previous experimental studies concerning TAR cotranscriptional folding were conducted using a relatively slow transcription speed of approximately 4 nt/s, as opposed to the in vivo transcription speed that ranges from around 8 nt/s during the initiation phase to an average of 40 nt/s (26,53). Genome-wide analysis of Pol II elongation dynamics has revealed that the average elongation rate of Pol II undergoes acceleration, transitioning from approximately 0.5 kb/min in the early elongation stage to 2.4 kb/min in the late stage (58). In order to investigate the influence of transcription speed on the TAR folding pathway, we conducted cotranscriptional folding simulations under different transcription speeds, namely 8, 20 and 40 nt/s (8,43).

**Figure 7.**
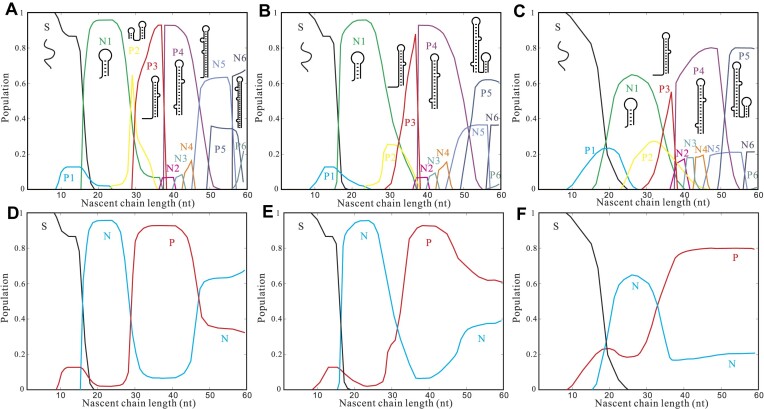
(A–C) Population kinetics during cotranscriptional folding at transcription speeds 8 nt/s (**A**), 20 nt/s (**B**) and 40 nt/s (**C**). P1–P4 and N1–N4 are the structures in the (non-native) pause-pathway and the native-pathway (see Figure 1), respectively. See Supplementary Figure S1 for the structures of P5, P6 and Supplementary Figure S2 for the structures of N5, N6. Illustrations for the structures with the highest population along the chain elongation are shown. (D–F) Population kinetics of the pause-pathway and the native-pathway at transcription speeds 8 nt/s (**D**), 20 nt/s (**E**) and 40 nt/s (**F**). P, N and S denote the pause-pathway, the native-pathway and the unfolded single-strand RNA, respectively.

Figures 7A-C illustrate the computational results, demonstrating the populations of different structures over time at transcription speeds of 8, 20 and 40 nt/s, respectively. Increasing the transcription speed from 4 to 8 nt/s does not alter the structure transitions with high transition rates (e.g., S to N1, P3 to P4). Moreover, the structural population remains largely unchanged. As depicted in Figure 7A, a higher transcription speed leads to a narrower time window for the transition from P4 to N4/N5, resulting in a reduced population flow from the pause-pathway to the native-pathway before P5 is formed. Furthermore, the formation of P5 significantly reduces the inter-pathway transitions towards the native-like structures (N4 and N5), because the newly formed helix in P5 (see Supplementary Figure S1) disrupts the pseudoknot-assisted transition from P4 to N4. Additionally, our computation suggests that at a nascent chain length of 44-nt, the population of the pause-pathway exceeds 80%. This proportion is approximately 10% higher in comparison to the slower transcription speed of 4 nt/s. At the end of transcription, the population of the native-pathway experiences a reduction of approximately 20% in comparison to the low-speed (4 nt/s) scenario.

For the faster transcription at 20 nt/s, as shown in Figure 7E, the population of the pause-pathway exceeds that of the native pathway in the final products. This outcome stands in contrast to the results obtained at lower speeds, as illustrated in Figure 2B, where the native pathway (N) is predominant in the final product. In the case of transcription speed at 40 nt/s, as shown in Figure 7F, the population of the pause-pathway maintains a population of approximately 80%, while the population flux for inter-pathway transitions from the pause-pathway to the native pathway is minimal.

The dependence of the pathway on transcription speed can be explained by the competition between nascent structure transitions and chain elongation. At high transcription speeds, such as 40 nt/s, the pseudoknot-assisted transition from the non-native intermediate P4 to the native-like TAR structure N4 becomes feasible only when the downstream segment of P4 (+40 to +44) is single-stranded and available for base-pairing. Additionally, if the nascent chain reaches +53 before the P4 to N4 transition occurs, the chain rapidly folds into the non-native intermediate P5. Within P5, nucleotides +40 to +44 form a stable helix through base-pairing, effectively preventing the pseudoknot-assisted transition from P4 to N4. Eventually, as illustrated in Supplementary Figure S1, the unfolding of a helix enables the transition of P5 and subsequent P6 structures towards the native TAR state. Our results agree with the experimental observations that initial elongation is slow because of the promoter proximal pausing of Pol II on TAR in cells.

### Effect of mutations on TAR folding pathway

The thermostability of cotranscriptional folding intermediates is crucial in determining the structure transitions. Therefore, mutations in TAR could alter the transition dynamics and, consequently, affect pausing kinetics. Experimental testing of various TAR mutants has been conducted. We examined the mutations studied in the previous experiments (26), and our calculations support the hypothesis that the P4 structure contributes to polymerase pausing, with the transition from P4 to N4 showing a positive correlation with the half-life of polymerase pausing. Subsequently, we examined whether TAR sequences in clinical samples exhibit similar cotranscriptional folding pathways and P4-to-N4 transition kinetics.

HIV-1 reverse transcriptase is known for its low fidelity, which contributes to the high mutation rate and facilitates rapid evolution and escape from antiviral drugs. These mutations can have varying effects on the biological adaptability of the virus, as they are often tolerable and can either enhance or diminish its adaptability. Specific mutations or polymorphisms in the sequence have the potential to influence the cotranscriptional folding pathway of TAR, which in turn regulates TAR-induced trans-activation of HIV-1. Because the non-native intermediate P4 plays a crucial role in the transcriptional pause at +62U, mutations that impact the stability of P4 and P4-related structural transitions can potentially alter the transcriptional folding pathway of TAR.

To investigate the effect of mutations on TAR folding pathway, we identified mutations or polymorphisms that could potentially increase the stability of P4, thereby possibly raising the energy barrier for the transition from P4 to N4. One such example is the U31A single mutation, which can form a base pair with U10 in P4 while not significantly affecting the free energy of N4. This mutation leads to a less favorable transition from P4 to N4. However, in our search for patient sequences carrying these specific mutations, we consistently observed the presence of additional mutations alongside the targeted mutation. As a result, we selected two HIV-1 mutant sequences that have been clinically reported and deposited in GenBank: the South Africa isolate (GenBank: DQ369978.1) and the Ghanaian isolate (GenBank: AB231894.1). While both isolates contain the U31A mutation to stabilize P4, the South Africa isolate includes additional G11A and U13G mutations, and the Ghanaian isolate has additional G11U, A48G, U50A, and A51G mutations. These additional mutations are predicted to either have no impact or negatively impact the thermostability of P4 and N4. Consequently, the energy barrier between P4 and N4, as well as their pausing half-life, is likely to remain similar to the wide type (HIV-1 strain NL4-3).

**Figure 8.**
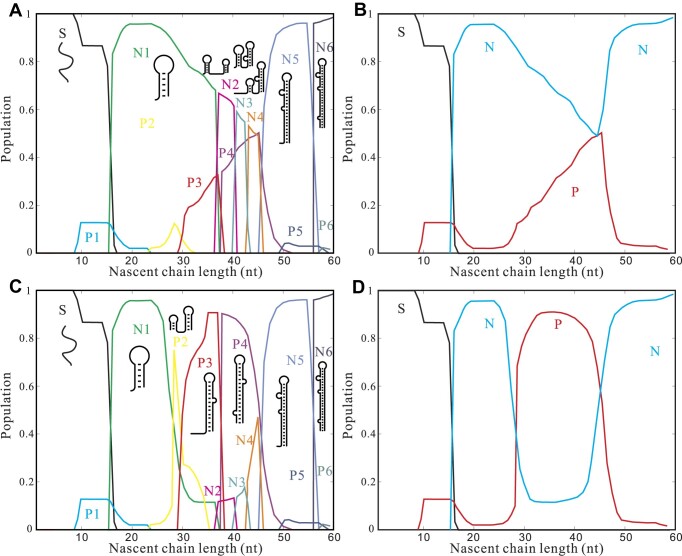
The populational kinetics during the cotranscriptional folding for South Africa mutant (**A**) and Ghanaian (**C**). Populations are calculated from Equation 1. Illustrations for the structures with the highest population along the chain elongation are shown. (**B**) and (**D**) show the population kinetics of the pause-pathway and the native-pathway for South Africa mutant (**B**) and Ghanaian (**D**), respectively. Populations are calculated from Equation (2) and pathways are labeled as P for the non-native pathway and N for the native-pathway, respectively. S denotes the unfolded state.

We first computed the folding free energy of TAR and all the possible folding intermediates for the South Africa and Ghanaian isolates. As illustrated in Supplementary Figure S4, the native TAR structure is marginally destabilized for the South Africa mutant (−28.9 kcal/mol) compared to the wild type (−32.9 kcal/mol), while the Ghanaian mutant maintains nearly the same stability (−32.5 kcal/mol). Given that RNA cotranscriptional folding is kinetically controlled, the overall folding pathway is determined by the stabilities of the intermediates and the free energy barriers in the structure transitions. To facilitate the comparison with the wild-type sequence, we assumed a transcription speed of 4 nt/s, consistent with the speed employed in the in vitro experiment (22,23). Our predictions indicate that the population of P4 at the pause site (transcription at +62U with 44 nucleotides available for folding), as depicted in Figure 8, decreases from approximately 75% for the wild type to around 50% for the South Africa mutant and approximately 55% for the Ghanaian mutant.

Through the computation of the folding free-energy landscape for the cotranscriptional folding pathway, we observed that for both the South Africa and Ghanaian isolates, the pause-inducing non-native intermediate P4 was equally destabilized by approximately 0.7 kcal/mol. The destabilization of the native folded TAR in the South Africa isolate did not exhibit a significant pathway shift. Indeed, at the pausing site, as depicted in Supplementary Figure S4, the competitive structures N2, N3 and N4 in the native-pathway remained unaffected by the mutations in both the South Africa and Ghanaian isolates. Consequently, the mutations in both the South Africa and Ghanaian isolates destabilized only the intermediates on the pause-pathway, resulting in a greater population flux into the native pathway.

Our computation further shows that the mutations present in both the South Africa and Ghanaian mutants can affect the stabilities of pseudoknots in the transition from P4 to N4, and these mutations have an impact on the overall pausing lifetime. We observed that the predicted pausing half-life for the Ghanaian mutant decreased from about 15 s to approximately 11 s, while the half-life for the South Africa mutant (14 s) showed a negligible change. In the case of the South Africa mutant, the pseudoknot PK6, which facilitates the P4 to N4 transition, is destabilized. Considering that the P4 itself is destabilized, the effective kinetic barrier and the transition rate for the transition from P4 to PK6 remains nearly unchanged. Simultaneously, the transition from PK6 to PK7 involves different base pair rearrangements, leading to a decreased transition rate from PK6 to PK7. However, since the mutations do not affect N4, there are no notable changes in the PK7 to N4 transition rate. As the PK7 to N4 transition is the rate-limiting step for the structure transition from P4 to N4, the mutation does not induce significant changes in the overall rate for the P4 to N4 transition. In contrast, the Ghanaian mutant exhibits a notable difference in the stability of PK7. This difference contributes to an increased transition rate from PK7 to N4, while the transition rates between P4, PK6, and PK7 remain relatively unchanged. As a result, there is an overall acceleration in the transition from P4 to N4 (see Supplementary Figure S5).

## Discussion

We have investigated HIV-1 TAR RNA cotranscriptional folding kinetics using a combined computational and NMR experimental approach. Through computational simulations, we identified two parallel cotranscriptional folding pathways: the pause-pathway associated with pause-inducing non-native TAR RNA structures, and the native-pathway for the folding of native TAR structures. The inter- and intra-pathway transitions are crucial in shaping the overall folding kinetics of TAR RNA and these transitions play a crucial role in defining the kinetic mechanisms associated with transcriptional pause, Tat-TAR mediated trans-activation, and the subsequent release from the pause state. Our computational predictions have been corroborated by NMR spectroscopy analysis of imino resonances, providing further support for the existence of cotranscriptional folding intermediates. We summarize the major findings as follows:

- At a slow transcription speed of 4 nt/s, the folding before reaching the pause site +62U is dominated by the pause-pathway, characterized by the formation of the non-native intermediate (P4 in Figure 1). Once the transcription surpasses +62U, the P4 population transitions to the native-like structure N4 through the native-pathway.
- The transition from the pause-pathway to the native-pathway (from P4 to N4) is facilitated by the formation of pseudoknot.
- Transcriptional pause can be induced by the presence of the non-native hairpin structures P4 (and also possibly P*). The existence of the pseudoknot-assisted transition from the non-native intermediate (P4) to the native-like structure (N4) enables the pause escaping.
- A higher transcription speed without pause fails to provide a sufficient time window for the transitions from the non-native intermediates (in the pause-pathway) to the native-like TAR structures (in the native-pathway). As a result, the non-native structures dominate the population in the transcription product.

To explore the RNA sequence variation on the TAR folding pathways, we investigate two HIV-1 sequences isolated from patients. We found that the mutation (U31A) altered structural stabilities of the folding intermediates, causing changes to the folding kinetics. However, the overall folding pathway, characterized by the formation of non-native intermediates and the ultimate dominance of the native TAR structure as the final product, closely resembles that of the wild-type sequence.

Located at the 5′-end of the HIV-1 RNA, TAR is the first functional RNA motif that is transcribed from integrated proviral DNA. Due to its critical role in recruiting cyclin-dependent kinase 9 to transactivate transcription (4,6,10–16) and stimulating cotranscriptional capping of viral RNA (73,74) the cotranscriptional folding pathway of TAR is expected to be tightly controlled. Indeed, analysis of two HIV clinical sequences revealed that the mutations or polymorphisms in TAR balance the thermostability of intermediates and transition rates between the intermediates. Our investigation revealed that the U31A mutation altered the thermostability of folding intermediates, causing changes in the folding kinetics. However, the overall folding pathway, characterized by the formation of non-native intermediates and the dominance of the native TAR structure in the final product, closely resembles that of the wild-type sequence. Similar structure population distribution (S, N and P, see Figure 2B) at critical RNA length were also observed. For example, cotranscriptional capping occurs when the nascent RNA reaches 19–22 nt (73), and the promoter proximal pausing happens at +62 nt (26). The S, N and P distribution at these lengths are similar among the WT (HIV-1 NL4-3) and the South Africa and Ghanaian isolates. Upon transcription initiation, Pol II elongation rate is generally slow and measured approximately 0.2–0.5 kb/min, and then accelerates to 2.5–3.2 kb/min (26,58,75,76). TAR is synthesized with a relatively slow rate, and therefore the folding intermediates revealed in our studies are likely to persist for an extended duration compared to those RNA cotranscriptional folding intermediates produced by a fast polymerase. As a result, these TAR cotranscriptional folding intermediates could potentially serve as drug targets that are previously overlooked and underestimated. Future directions involve using methods such as NMR to capture the high-resolution three-dimensional structures of RNAs along the cotranscriptional folding pathway and screening drug molecules targeting the RNA structures (46,77).

The 5′- to 3′-folding polarity of nascently synthesized RNA produces many transient RNA conformations that lack the opportunity to reach thermodynamic equilibrium before they are captured by nearby protein and nucleic acid factors for important RNA processing steps. Consequently, mapping the RNA cotranscriptional folding pathway is valuable for identifying these transient thermodynamically unstable structures and elucidating how these kinetic structures contribute to gene expression. Studies of bacterial riboswitch RNAs have deciphered the cotranscriptional folding pathways in the ligand-induced ON or OFF states of transcription (5–9). While many factors may influence cotranscriptional RNA folding (10), polymerase rate emerges as a critical determinant in modulating the ‘nascent RNA structurome’ in eukaryotic cells (11). Employing a combined chemical and enzymatic probing approach, the Bentley group demonstrated distinct RNA structural changes by a slow Pol II mutant, influencing widespread RNA processing (1). Recent findings in a variety of species have documented average speed of Pol II accelerates with age, contributing to the dysregulation of proteomes in aging adults (2). Such intricate RNA folding regulation by the speed of Pol II plays a central role in the adaptation, development and overall fitness of organisms (1–4). Transcription speed control is an important factor for cotranscriptional folding pathway predictions. Even though we are using an average transcription speed in the present work, it is feasible to convert the constant transcription speed into a function of sequence content when there is adequate information on how specific sequences influence the regulation of polymerase speed. The LZ model developed in this study represents an initial step towards unraveling the parallel cotranscriptional folding pathways of RNA, which will significantly contribute to our comprehension of how nascent RNA structurome determines the proteome in cells.

The current model, however, has its limitations. First, the current model evaluates the free energies based on 2D structures. As a result, the model cannot explicitly account for 3D interactions, such as steric impediment from the RNAP II and other RNA-polymerase interactions. Second, the current folding model does not consider long-range kissing interactions. Such interactions could be important for the folding of large RNA structures, although such interactions are unlikely important for the short HIV-1 TAR RNA in the present study. Third, the current model does not explicitly account for the metal ions effect such as the effect of Mg^2+^ binding, which could be crucial for the stabilization of RNA tertiary folds. With the current study as a robust starting point, future development of the model should address the above issues.

## Supplementary Material

gkae362_Supplemental_File

## Data Availability

The data are available in the article and the online supplementary material. The LZ code is available in Figshare at: https://doi.org/10.6084/m9.figshare.25668729.

## References

[1] Coffin J.M., Hughes S.H., Varmus H.E. Retroviruses. 1997; NY Cold Spring Habour Laboratory Press. 21433340

[2] Bevilacqua P.C., Tolbert B.S. Regulatory mechanisms through RNA conformational switching and dynamics. J. Mol. Biol. 2022; 434:167794. 35988750 10.1016/j.jmb.2022.167794 PMC9484478

[3] Wu M.T.P., D’Souza V. Alternate RNA structures. Cold Spring Harb. Perspect. Biol. 2020; 12:a032425. 31896543 10.1101/cshperspect.a032425 PMC6942119

[4] Bannwarth S., Gatignol A. HIV-1 TAR RNA: the target of molecular interactions between the virus and its host. Curr. HIV Res. 2005; 3:61–71. 15638724 10.2174/1570162052772924

[5] Schulze-Gahmen U., Hurley J.H. Structural mechanism for HIV-1 TAR loop recognition by Tat and the super elongation complex. Proc. Natl. Acad. Sci. U.S.A. 2018; 115:12973–12978. 30514815 10.1073/pnas.1806438115 PMC6305006

[6] Davidson A., Leeper T.C., Athanassiou Z., Patora-Komisarska K., Karn J., Robinson J.A., Varani G. Simultaneous recognition of HIV-1 TAR RNA bulge and loop sequences by cyclic peptide mimics of Tat protein. Proc. Natl. Acad. Sci. U.S.A. 2009; 106:11931–11936. 19584251 10.1073/pnas.0900629106 PMC2715490

[7] Roy S., Delling U., Chen C.H., Rosen C.A., Sonenberg N. A bulge structure in HIV-1 TAR RNA is required for Tat binding and Tat-mediated trans-activation. Genes Dev. 1990; 4:1365–1373. 2227414 10.1101/gad.4.8.1365

[8] Cullen B.R. Trans-activation of human immunodeficiency virus occurs via a bimodal mechanism. Cell. 1986; 46:973–982. 3530501 10.1016/0092-8674(86)90696-3

[9] Pham V.V., Salguero C., Khan S.N., Meagher J.L., Brown W.C., Humbert N., de Rocquigny H., Smith J.L., D’Souza V.M. HIV-1 Tat interactions with cellular 7SK and viral TAR RNAs identifies dual structural mimicry. Nat. Commun. 2018; 9:4266. 30323330 10.1038/s41467-018-06591-6 PMC6189040

[10] Cullen B.R. Regulation of HIV-1 gene expression. FASEB J. 1991; 5:2361–2368. 1712325 10.1096/fasebj.5.10.1712325

[11] Karn J., Stoltzfus C.M. Transcriptional and posttranscriptional regulation of HIV-1 gene expression. Cold Spring Harb. Perspect. Med. 2012; 2:a006916. 22355797 10.1101/cshperspect.a006916 PMC3281586

[12] Ouellet D.L., Vigneault-Edwards J., Létourneau K., Gobeil L.A., Plante I., Burnett J.C., Rossi J.J., Provost P. Regulation of host gene expression by HIV-1 TAR microRNAs. Retrovirology. 2013; 10:86. 23938024 10.1186/1742-4690-10-86 PMC3751525

[13] Roebuck K.A., Saifuddin M. Regulation of HIV-1 transcription. Gene Expression. 1999; 8:67–84. 10551796 PMC6157391

[14] Aboul-ela F., Karn J., Varani G. Structure of HIV-1 TAR RNA in the absence of ligands reveals a novel conformation of the trinucleotide bulge. Nucleic Acids Res. 1996; 24:3974–3981. 8918800 10.1093/nar/24.20.3974 PMC146214

[15] Zhang J., Tamilarasu N., Hwang S., Garber M.E., Huq I., Jones K.A., Rana T.M. HIV-1 TAR RNA enhances the interaction between Tat and cyclin T1. J. Biol. Chem. 2000; 275:34314–34319. 10944537 10.1074/jbc.M006804200

[16] Unwalla H.J., Li M.J., Kim J.D., Li H.T., Ehsani A., Alluin J., Rossi J.J. Negative feedback inhibition of HIV-1 by TAT-inducible expression of siRNA. Nat. Biotechnol. 2004; 22:1573–1578. 15568018 10.1038/nbt1040

[17] Guenther M.G., Levine S.S., Boyer L.A., Jaenisch R., Young R.A. A chromatin landmark and transcription initiation at most promoters in human cells. Cell. 2007; 130:77–88. 17632057 10.1016/j.cell.2007.05.042 PMC3200295

[18] Core L.J., Waterfall J.J., Lis J.T. Nascent RNA sequencing reveals widespread pausing and divergent initiation at human promoters. Science. 2008; 322:1845–1848. 19056941 10.1126/science.1162228 PMC2833333

[19] Muse G.W., Gilchrist D.A., Nechaev S., Shah R., Parker J.S., Grissom S.F., Zeitlinger J., Adelman K. RNA polymerase is poised for activation across the genome. Nat. Genet. 2007; 39:1507–1511. 17994021 10.1038/ng.2007.21 PMC2365887

[20] Core L., Adelman K. Promoter-proximal pausing of RNA polymerase II: a nexus of gene regulation. Genes Dev. 2019; 33:960–982. 31123063 10.1101/gad.325142.119 PMC6672056

[21] Saunders A., Core L.J., Sutcliffe C., Lis J.T., Ashe H.L. Extensive polymerase pausing during Drosophila axis patterning enables high-level and pliable transcription. Genes Dev. 2013; 27:1146–1158. 23699410 10.1101/gad.215459.113 PMC3672648

[22] Lagha M., Bothma J.P., Esposito E., Ng S., Stefanik L., Tsui C., Johnston J., Chen K., Gilmour D.S., Zeitlinger J. et al. Paused Pol II coordinates tissue morphogenesis in the Drosophila embryo. Cell. 2013; 153:976–987. 23706736 10.1016/j.cell.2013.04.045 PMC4257494

[23] Guo S., Yamaguchi Y., Schilbach S., Wada T., Lee J., Goddard A., French D., Handa H., Rosenthal A. A regulator of transcriptional elongation controls vertebrate neuronal development. Nature. 2000; 408:366–369. 11099044 10.1038/35042590

[24] Williams L.H., Fromm G., Gokey N.G., Henriques T., Muse G.W., Burkholder A., Fargo D.C., Hu G., Adelman K. Pausing of RNA polymerase II regulates mammalian developmental potential through control of signaling networks. Mol. Cell. 2015; 58:311–322. 25773599 10.1016/j.molcel.2015.02.003 PMC4402150

[25] Bahat A., Lahav O., Plotnikov A., Leshkowitz D., Dikstein R. Targeting Spt5-Pol II by small-molecule inhibitors uncouples distinct activities and reveals additional regulatory roles. Mol. Cell. 2019; 76:617–631. 31564557 10.1016/j.molcel.2019.08.024

[26] Palangat M., Meier T.I., Keene R.G., Landick R. Transcriptional pausing at+ 62 of the HIV-1 nascent RNA modulates formation of the TAR RNA structure. Mol. Cell. 1998; 1:1033–1042. 9651586 10.1016/s1097-2765(00)80103-3

[27] Yonaha M., Proudfoot N.J. Specific transcriptional pausing activates polyadenylation in a coupled in vitro system. Mol. Cell. 1999; 3:593–600. 10360175 10.1016/s1097-2765(00)80352-4

[28] Park N.J., Tsao D.C., Martinson H.G. The two steps of poly (A)-dependent termination, pausing and release, can be uncoupled by truncation of the RNA polymerase II carboxyl-terminal repeat domain. Mol. Cell. Biol. 2004; 24:4092–4103. 15121832 10.1128/MCB.24.10.4092-4103.2004 PMC400489

[29] de la Mata M., Alonso C.R., Kadener S., Fededa J.P., Blaustein M., Pelisch F., Cramer P., Bentley D., Kornblihtt A.R. A slow RNA polymerase II affects alternative splicing in vivo. Mol. Cell. 2003; 12:525–532. 14536091 10.1016/j.molcel.2003.08.001

[30] Kireeva M.L., Hancock B., Cremona G.H., Walter W., Studitsky V.M., Kashlev M. Nature of the nucleosomal barrier to RNA polymerase II. Mol. Cell. 2005; 18:97–108. 15808512 10.1016/j.molcel.2005.02.027

[31] Chiu L.Y., Emery A., Jain N., Sugarman A., Kendrick N., Luo L., Ford W., Swanstrom R., Tolbert B.S. Encoded conformational dynamics of the HIV splice site A3 regulatory locus: implications for differential binding of hnRNP splicing auxiliary factors. J. Mol. Biol. 2022; 434:167728. 35870649 10.1016/j.jmb.2022.167728 PMC9945881

[32] Boyle J., Robillard G.T., Kim S.H. Sequential folding of transfer RNA: a nuclear magnetic resonance study of successively longer tRNA fragments with a common 5′ end. J. Mol. Biol. 1980; 139:601–625. 6997498 10.1016/0022-2836(80)90051-0

[33] Kramer F.R., Mills D.R. Secondary structure formation during RNA synthesis. Nucleic Acids Res. 1981; 9:5109–5124. 6171773 10.1093/nar/9.19.5109 PMC327502

[34] Pan T., Sosnick T. RNA folding during transcription. Annu. Rev. Biophys. Biomol. Struct. 2006; 35:161–175. 16689632 10.1146/annurev.biophys.35.040405.102053

[35] Frieda K.L., Block S.M. Direct observation of cotranscriptional folding in an adenine riboswitch. Science. 2012; 338:397–400. 23087247 10.1126/science.1225722 PMC3496414

[36] Incarnato D., Morandi E., Anselmi F., Simon L.M., Basile G., Oliviero S. In vivo probing of nascent RNA structures reveals principles of cotranscriptional folding. Nucleic Acids Res. 2017; 45:9716–9725. 28934475 10.1093/nar/gkx617 PMC5766169

[37] Helmling C., Wacker A., Wolfinger M.T., Hofacker I.L., Hengesbach M., Fürtig B., Schwalbe H. NMR structural profiling of transcriptional intermediates reveals riboswitch regulation by metastable RNA conformations. J. Am. Chem. Soc. 2017; 139:2647–2656. 28134517 10.1021/jacs.6b10429

[38] Zamft B., Bintu L., Ishibashi T., Bustamante C. Nascent RNA structure modulates the transcriptional dynamics of RNA polymerases. Proc. Natl. Acad. Sci. U.S.A. 2012; 109:8948–8953. 22615360 10.1073/pnas.1205063109 PMC3384149

[39] Larsson A.J., Johnsson P., Hagemann-Jensen M., Hartmanis L., Faridani O.R., Reinius B., Segerstolpe Å., Rivera C.M., Ren B., Sandberg R. Genomic encoding of transcriptional burst kinetics. Nature. 2019; 565:251–254. 30602787 10.1038/s41586-018-0836-1 PMC7610481

[40] Hidalgo L., Swanson C.M. Regulation of human immunodeficiency virus type 1 (HIV-1) mRNA translation. Biochem. Soc. Trans. 2017; 45:353–364. 28408475 10.1042/BST20160357

[41] Schroeder S.J. Perspectives on viral RNA genomes and the RNA folding problem. Viruses. 2020; 12:1126. 33027988 10.3390/v12101126 PMC7600889

[42] Watters K.E., Strobel E.J., Yu A.M., Lis J.T., Lucks J.B. Cotranscriptional folding of a riboswitch at nucleotide resolution. Nat. Struct. Mol. Biol. 2016; 23:1124–1131. 27798597 10.1038/nsmb.3316 PMC5497173

[43] Tadigotla V.R., Maoiléidigh D.Ó., Sengupta A.M., Epshtein V., Ebright R.H., Nudler E., Ruckenstein A.E. Thermodynamic and kinetic modeling of transcriptional pausing. Proc. Natl. Acad. Sci. U.S.A. 2006; 103:4439–4444. 16537373 10.1073/pnas.0600508103 PMC1450190

[44] Aitken S., Alexander R.D., Beggs J.D. Modelling reveals kinetic advantages of cotranscriptional splicing. PLoS Comput. Biol. 2011; 7:e1002215. 22022255 10.1371/journal.pcbi.1002215 PMC3192812

[45] Honkela A., Peltonen J., Topa H., Charapitsa I., Matarese F., Grote K., Stunnenberg H.G., Reid G., Lawrence N.D., Rattray M. Genome-wide modeling of transcription kinetics reveals patterns of RNA production delays. Proc. Natl. Acad. Sci. U.S.A. 2015; 112:13115–13120. 26438844 10.1073/pnas.1420404112 PMC4620908

[46] Angela M.Y., Gasper P.M., Cheng L., Lai L.B., Kaur S., Gopalan V., Chen A.A., Lucks J.B. Computationally reconstructing cotranscriptional RNA folding from experimental data reveals rearrangement of non-native folding intermediates. Mol. Cell. 2021; 81:870–883. 33453165 10.1016/j.molcel.2020.12.017 PMC8061711

[47] Xu X., Jin L., Xie L., Chen S.J. Landscape zooming toward the prediction of RNA cotranscriptional folding. J. Chem. Theory Comput. 2022; 18:2002–2015. 35133833 10.1021/acs.jctc.1c01233 PMC8904291

[48] Schroeder S.J. Challenges and approaches to predicting RNA with multiple functional structures. RNA. 2018; 24:1615–1624. 30143552 10.1261/rna.067827.118 PMC6239171

[49] Richter S.N., Belanger F., Zheng P., Rana T.M. Dynamics of nascent mRNA folding and RNA–protein interactions: an alternative TAR RNA structure is involved in the control of HIV-1 mRNA transcription. Nucleic Acids Res. 2006; 34:4278–4292. 16920743 10.1093/nar/gkl499 PMC1616951

[50] Cao S., Chen S.J. Predicting RNA folding thermodynamics with a reduced chain representation model. RNA. 2005; 11:1884–1897. 16251382 10.1261/rna.2109105 PMC1370876

[51] Cao S., Chen S.J. Predicting structures and stabilities for H-type pseudoknots with interhelix loops. RNA. 2009; 15:696–706. 19237463 10.1261/rna.1429009 PMC2661829

[52] Cheng Y., Zhang S., Xu X., Chen S.J. Vfold2D-MC: a physics-based hybrid model for predicting RNA secondary structure folding. J. Phys. Chem. B. 2021; 125:10108–10118. 34473508 10.1021/acs.jpcb.1c04731 PMC8903033

[53] Buratowski S. The basics of basal transcription by RNA polymerase II. Cell. 1994; 77:1–3. 10.1016/0092-8674(94)90226-7 8156586

[54] Landick R. RNA polymerase slides home: pause and termination site recognition. Cell. 1997; 88:741–744. 9118216 10.1016/s0092-8674(00)81919-4

[55] Jones K.A. Taking a new TAK on tat transactivation. Genes Dev. 1997; 11:2593–2599. 9334323 10.1101/gad.11.20.2593

[56] Berkhout B., Silverman R.H., Jeang K.T. Tat trans-activates the human immunodeficiency virus through a nascent RNA target. Cell. 1989; 59:273–282. 2478293 10.1016/0092-8674(89)90289-4

[57] Boireau S., Maiuri P., Basyuk E., de la Mata M., Knezevich A., Pradet-Balade B., Bäcker V., Kornblihtt A., Marcello A., Bertrand E. The transcriptional cycle of HIV-1 in real-time and live cells. J. Cell Biol. 2007; 179:291–304. 17954611 10.1083/jcb.200706018 PMC2064765

[58] Jonkers I., Kwak H., Lis J.T. Genome-wide dynamics of Pol II elongation and its interplay with promoter proximal pausing, chromatin, and exons. eLife. 2014; 3:e02407. 24843027 10.7554/eLife.02407 PMC4001325

[59] Danko C.G., Hah N., Luo X., Martins A.L., Core L., Lis J.T., Siepel A., Kraus W.L. Signaling pathways differentially affect RNA polymerase II initiation, pausing, and elongation rate in cells. Mol. Cell. 2013; 50:212–222. 23523369 10.1016/j.molcel.2013.02.015 PMC3640649

[60] Fuchs G., Voichek Y., Benjamin S., Gilad S., Amit I., Oren M. 4sUDRB-seq: measuring genomewide transcriptional elongation rates and initiation frequencies within cells. Genome Biol. 2014; 15:R69. 24887486 10.1186/gb-2014-15-5-r69 PMC4072947

[61] Veloso A., Kirkconnell K.S., Magnuson B., Biewen B., Paulsen M.T., Wilson T.E., Ljungman M. Rate of elongation by RNA polymerase II is associated with specific gene features and epigenetic modifications. Genome Res. 2014; 24:896–905. 24714810 10.1101/gr.171405.113 PMC4032854

[62] Gregersen L.H., Mitter R., Svejstrup J.Q. Using TTchem-seq for profiling nascent transcription and measuring transcript elongation. Nat. Protoc. 2020; 15:604–627. 31915390 10.1038/s41596-019-0262-3

[63] Isambert H., Siggia E.D. Modeling RNA folding paths with pseudoknots: application to hepatitis delta virus ribozyme. Proc. Natl. Acad. Sci. U.S.A. 2000; 97:6515–6520. 10823910 10.1073/pnas.110533697 PMC18642

[64] Shapiro B.A., Bengali D., Kasprzak W., Wu J.C. RNA folding pathway functional intermediates: their prediction and analysis. J. Mol. Biol. 2001; 312:27–44. 11545583 10.1006/jmbi.2001.4931

[65] Xu X., Chen S.J. Kinetic mechanism of conformational switch between bistable RNA hairpins. J. Am. Chem. Soc. 2012; 134:12499–12507. 22765263 10.1021/ja3013819 PMC3427750

[66] Artsimovitch I., Landick R. Interaction of a nascent RNA structure with RNA polymerase is required for hairpin-dependent transcriptional pausing but not for transcript release. Genes Dev. 1998; 12:3110–3122. 9765211 10.1101/gad.12.19.3110 PMC317188

[67] Landick R. The regulatory roles and mechanism of transcriptional pausing. Biochem. Soc. Trans. 2006; 34:1062–1066. 17073751 10.1042/BST0341062

[68] Milligan J.F., Uhlenbeck O.C. [5] Synthesis of small RNAs using T7 RNA polymerase. Meth. Enzymol. 1989; 180:51–62. 10.1016/0076-6879(89)80091-6 2482430

[69] Delaglio F., Grzesiek S., Vuister G.W., Zhu G., Pfeifer J., Bax A.D. NMRPipe: a multidimensional spectral processing system based on UNIX pipes. J. Biomol. NMR. 1995; 6:277–293. 8520220 10.1007/BF00197809

[70] Johnson B.A. Using NMRView to visualize and analyze the NMR spectra of macromolecules. Protein NMR Tech. 2004; 278:313–352. 10.1385/1-59259-809-9:313 15318002

[71] Gu W., Wind M., Reines D. Increased accommodation of nascent RNA in a product site on RNA polymerase II during arrest. Proc. Natl. Acad. Sci. U.S.A. 1996; 93:6935–6940. 8692922 10.1073/pnas.93.14.6935 PMC38912

[72] Muniz L., Nicolas E., Trouche D. RNA polymerase II speed: a key player in controlling and adapting transcriptome composition. EMBO J. 2021; 40:e105740. 34254686 10.15252/embj.2020105740 PMC8327950

[73] Chiu Y.L., Ho C.K., Saha N., Schwer B., Shuman S., Rana T.M. Tat stimulates cotranscriptional capping of HIV mRNA. Mol. Cell. 2002; 10:585–597. 12408826 10.1016/s1097-2765(02)00630-5

[74] Zhou M., Deng L., Kashanchi F., Brady J.N., Shatkin A.J., Kumar A. The Tat/TAR-dependent phosphorylation of RNA polymerase II C-terminal domain stimulates cotranscriptional capping of HIV-1 mRNA. Proc. Natl. Acad. Sci. U.S.A. 2003; 100:12666–12671. 14569024 10.1073/pnas.1835726100 PMC240675

[75] Baluapuri A., Hofstetter J., Stankovic N.D., Endres T., Bhandare P., Vos S.M., Adhikari B., Schwarz J.D., Narain A., Vogt M. et al. MYC recruits SPT5 to RNA polymerase II to promote processive transcription elongation. Mol. Cell. 2019; 74:674–687. 30928206 10.1016/j.molcel.2019.02.031 PMC6527870

[76] Liang K., Smith E.R., Aoi Y., Stoltz K.L., Katagi H., Woodfin A.R., Rendleman E.J., Marshall S.A., Murray D.C., Wang L. et al. Targeting processive transcription elongation via SEC disruption for MYC-induced cancer therapy. Cell. 2018; 175:766–779. 30340042 10.1016/j.cell.2018.09.027 PMC6422358

[77] Berman K.E., Steans R., Hertz L.M., Lucks J.B. A transient intermediate RNA structure underlies the regulatory function of the *E. coli* thiB TPP translational riboswitch. RNA. 2023; 29:1658–1672. 37419663 10.1261/rna.079427.122 PMC10578472

