# Supplementary material for: Kinetic pathway of HIV-1 TAR cotranscriptional folding
Source: Nucleic Acids Res. 2024 May 13;52(10):6066–78. doi: 10.1093/nar/gkae362 (PMC11162800; doi:10.1093/nar/gkae362)
Supplement: gkae362_Supplemental_File [file gkae362_supplemental_file.pdf]

Supplementary Information:

Kinetic pathway of HIV-1 TAR cotranscriptional folding

Lei Jin[1], Sicheng Zhang[1], Zhenwei Song[2], Xiao Heng[2,*], Shi-Jie Chen[1,2,*]
[1]Department of Physics and Institute of Data Science and Informatics, University of Missouri, Columbia, MO 65211, USA
[2] Department of Biochemistry, University of Missouri, Columbia, MO 65211, USA

Co-transcriptional folding model

Helix pools and structure partitioning

For a given RNA sequence, we first build a helix pool consisting of all the possible helices formed by canonical base pairs (A-U, G-C, and G-U). Each helix in the helix pool contains at least one base stack formed by two consecutive base pairs. Only saturated helices, which cannot be further extended on either side through canonical base pairs, are included in the helix pool. The free energy of each helix is evaluated by the base stacking energies from the Turner parameters [**?**]. In general, the size of the helix pool for a given RNA sequence grows rapidly with RNA sequence length.

RNA conformation partitions

We rank all the helices in the pool according to their free energies and select a given number of top-ranked lowest-free energy helices. We use the selected lowest-free energy helices to divide the RNA conformational space into different partitions. At this time, structures containing overlapping nucleotides (nucleotides belonging to two or more helices) or cross-linked base pairs such as base pairs in a pseudoknot are removed. We calculate the free energy of each partition using the Vfold2D model [**?**], which implements the dynamic programming algorithm to exhaustively sample the 2D structures with the helix constraints and applies the Turner parameters [**?**] and the Vfold-derived RNA loop parameters to evaluate the free energy of each sampled structure,where all the compatible stable and less-stable helices (helices not selected for the construction of partitions) are sampled.

Transition rates for helix-based kinetic moves

In general, the formation and disruption of stable helices usually involve a high kinetic barrier. The model uses stable helices to define partitions, assuming that structure transitions within each partition are faster than those between partitions. Therefore, each partition can be treated as a quasi-equilibrium macrostate, and the overall folding kinetics is mainly determined by the kinetic network of inter-partition transitions.

From the definition of partitions, different partitions differ by at least one helix and an inter-partition transition can be described as the deletion and addition of a helix ("helix-based transitions"). To compute the inter-partition kinetic rates, we consider all the possible kinetic pathways for the deletion/addition of a helix. On each pathway, structures are connected by the addition/deletion of a single base pair and the intermediate structures contain partially formed helix. We apply the conventional Metropolis rule [**?**] to calculate the rate constant k_{AB} for the transition from structure A to its neighboring structure B (through the addition/deletion of a base pair):

$$k_{AB} = k_0 \min\left(1, e^{-\Delta G_{AB}/k_B T}\right) \tag{1}$$

where, ΔG_{AB} = G_B - G_A is the free energy difference between the two states, k_B is the Boltzmann constant, T is the folding temperature, and k_0 is the solution-dependent attempt frequency to be determined from comparisons with experiment. Here we use the Vfold2D model to calculate the free energy of a structure. From the above constant k_{AB} for a base pair, we apply the KMC simulations [**?**, **?**] to calculate the rate for the whole helix from the pathways. the KMC simulation starts from the initial partition (before and the folding trajectory terminates once the final partitionis visited. We sample 5000 such trajectories, each of which gives a first passage time (FPT) from the initial helix-open partition to the final helix-close partition. The transition rate for the helix formation (addition) is calculated from the mean first passage time T_{FPT} averaged over all the 5000 trajectories as k^{+}_{helix} = 1 / T_{FPT}. The transition rate for helix disruption is calculated from the detailed balance condition as k^{-}_{helix} = $e^{-(G^{-}_{\mathrm{helix}} - G^{+}_{\mathrm{helix}})/k_B T} k^{+}_{\mathrm{helix}}$, where, G^{-}_{helix} and G^{+}_{helix} are the free energies of the helix-open and the helix-close partitions, respectively. We note that 5000 trajectories

are sufficient for the convergence of $T_{\rm FPT}$ according to our previous tests for various RNA hairpin and pseudoknots [**?**].

Master Equations and populational kinetics

From the inter-partition rate constants, we predict the detailed populational kinetics for each partition from the master equation. Specifically, the fractional population $p_i(t)$ of partition i (= 1, 2, $\cdots$, Ω, where Ω is the total number of partitions) evolves with time t according to the following master equation:

$$\frac{dp_i(t)}{dt} = \sum_j [k_{ji}p_j(t) - k_{ij}p_i(t)], \tag{2}$$

where, k_{ij} and k_{ji} are the rates for the transitions from partitions i to j and from j to i, respectively. The rate constants constitute an $\Omega \times \Omega$ rate matrix **M**, such that Eq. **??** has an equivalent matrix form of $\frac{d\mathbf{p}(t)}{dt}$ = $\mathbf{M} \cdot \mathbf{p}(t)$, with $\mathbf{M}_{ij}$ = k_{ji} for $i \neq j$, $\mathbf{M}_{ii}$ = - $\sum_{j \neq i} k_{ij}$, and **p**(t) the fractional populational vector of $[p_1(t)$, $p_2(t)$, $\cdots$, $p_\Omega(t)]$. Mathematically, by solving the eigenvalues λ_m and eigenvectors $\mathbf{n}_m$ (m = 1, 2, $\cdots$, Ω) of the rate matrix **M**, the population kinetics can be obtained analytically as

$$\mathbf{p}(t) = \sum_m C_m \mathbf{n}_m e^{\lambda_m t} \tag{3}$$

for the population of all the partitions at any time t, where C_m is a coefficient determined by the initial condition.

Co-transcriptional folding and transcription speed

Compared with the folding of a full-length sequence, RNA cotranscriptional folding involves the elongation of the RNA chain, resulting in a time-varying folding landscape during transcription. Therefore, the modeling of cotranscriptional folding requires iterative, stepwise simulations as explained below. Before the emerging of the (n+1)-th nucleotide (nt), the RNA chain of n nucleotides navigates the network of the n-nt partitions, with the initial population distribution inherited from the folding of the (n-1)-nt chain. As the (n+1)-th nucleotide is synthesized, the folding landscapes may undergo a significant change. At the time of a new nucleotide emerges, the helix pool and the resultant partitions are updated. As a result, the nascent chain explores a new ensemble of partitions and navigates a new energy landscape, accordingly, the stability of each partition, inter-partition transition rates, the eigenvalues and eigenvectors of the rate matrix **M**, and the initial condition-dependent coefficient C_m in Eq. **??** all need to be updated.

In this study, the whole cotranscriptional folding simulation is divided into a series of time windows. In the n-th time window with the n-th nucleotide newly transcribed (n = 1, 2, $\cdots$,N, where N is the total length of RNA), the folding of the (n-1)-nt chain L_{n-1} is terminated and the folding of the nascent n-nt chain L_n is started. We update the helix pool for L_n, from which we update the partitions. To investigate the folding of L_n, we need to determine the (initial) population of each L_n partition. We transfer the population distribution of L_{n-1} to that of L_n based on the relationship between two helix pools. The transcription of the nascent n-th nucleotide can lead to three types of structure changes: (1) no structural changes except that the 3' dangling end is elongated by one nucleotide; (2) a new lone base stack (two consecutive base pairs) is formed by three originally unpaired nucleotides and the newly added nucleotide; (3) a new base pair is added to an existing helix.
Because the above three types of structure changes are fast compared with RNA transcription, we assume local equilibrium between the (sub)structures of a partition can be quickly established, which allows us to use Boltzmann distribution to reassign the fractional population of each L_{n-1} partition to individual L_n structures.

In the theoretical calculation, we assume that the transcription rate is constant with a duration ΔT for each time window. ΔT can be treated as the average time duration for a new nucleotide to be transcribed and added to the nascent chain (also defined in the section of "Cotranscriptional folding on a variable landscape"). The time duration ΔT corresponds to the transcription speed of $(\Delta T)^{-1}$ nucleotides per unit time. All the calculations are carried out with temperature at 310 K, and k_0 = 1. Therefore, the simulation time scale is in the unit of k_0^{-1}, where k_0 is the prefactor in Eq. **??**.

In general, the transcription speed ranges from 200 nt/s in phages to 20-80 nt/s in bacteria and 10-20 nt/s in human polymerase. To convert the simulation timescale into a physical timescale, the solution-dependent attempt frequency k_0 is determined by the comparisons with (case-by-case) experimental data. For a typical value of 10^6 for k_0[**?**, **?**], the time duration $\Delta T \approx 10^5$ matches the transcription speed in human polymerase.

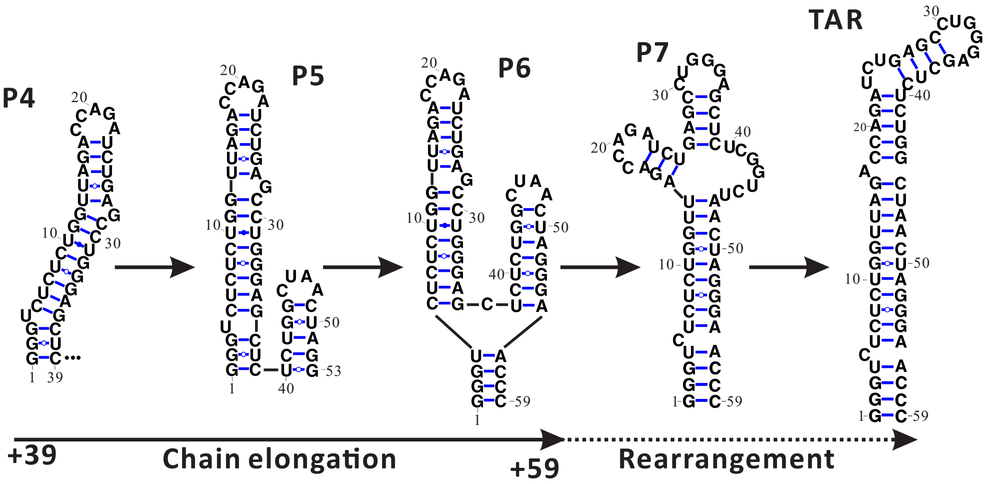

Figure S1: Co-transcriptional folding pathway from +39 to +59 (P4 to TAR) without pseudoknotes. Note P7 is an intermediate structure with insufficient population to be shown in Fig. 2.

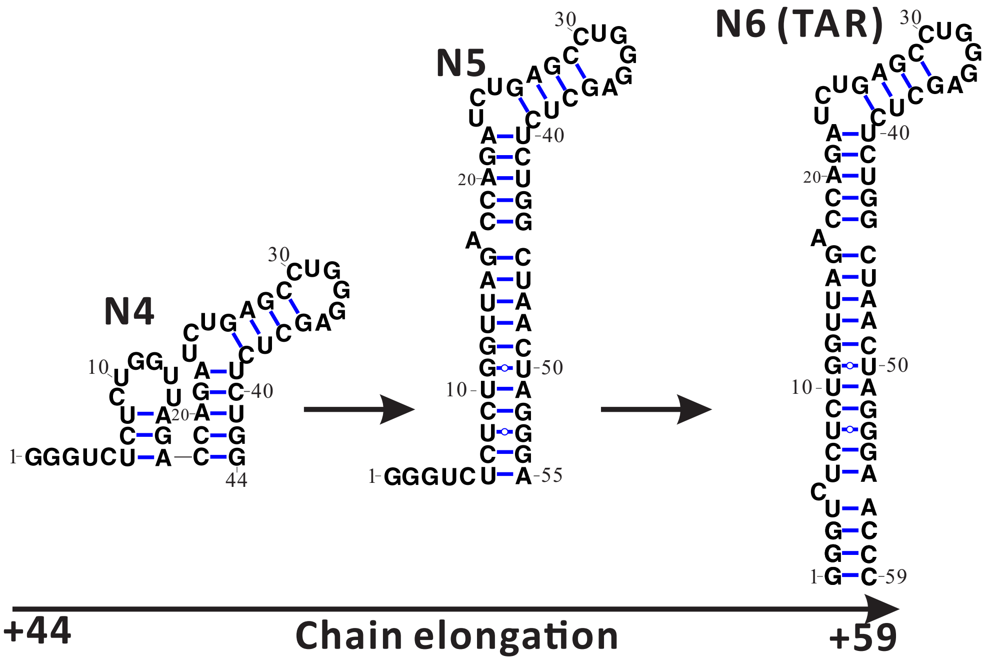

Figure S2: Co-transcriptional folding pathway from +44 to +59 (N4 to TAR). TAR is shown as N6 in Fig 2.

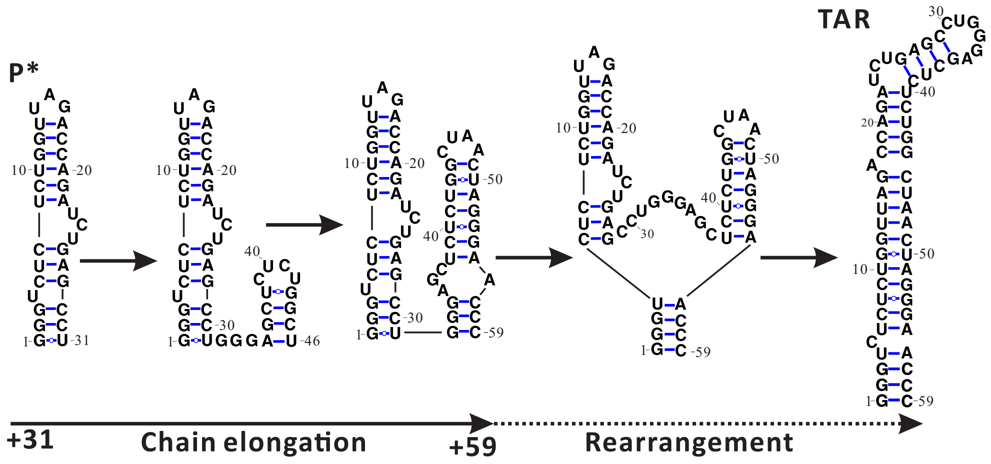

Figure S3: The co-transcriptional folding pathway from +31 to +59 (P* to TAR) without pseudoknots.

WT	GGGUCUCUCUGGUUAGACCAGAUCUGAGCCUGGGAGCUCUCUGGCUAACUAGGGAACCC
S Africa	GGGUCUCUCUAGGUAGACCAGAUCUGAGCCAGGGAGCUCUCUGGCUAACUAGGGAACCC
Ghanaian	GGGUCUCUCUUGUUAGACCAGAUCUGAGCCAGGGAGCUCUCUGGCUAGCAGGGGAACCC

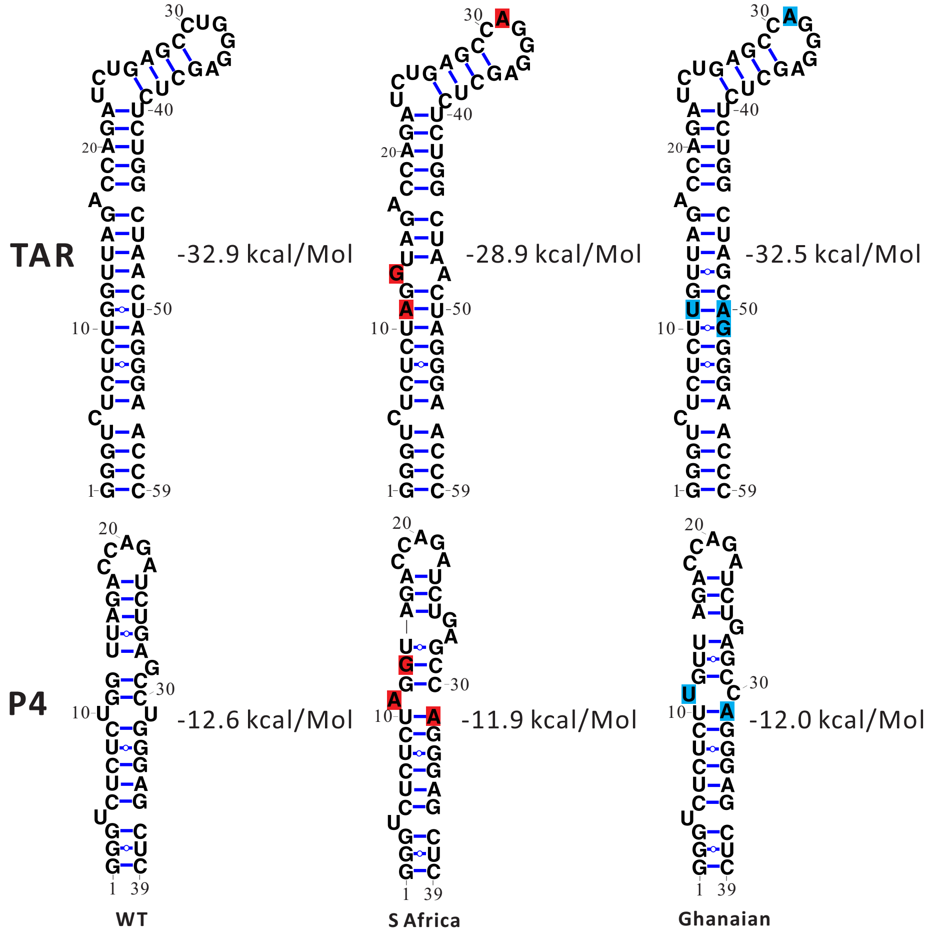

Figure S4: Sequence alignments for the mutants and predicted 2D structures for the TAR and misfolded intermediate structure P4.

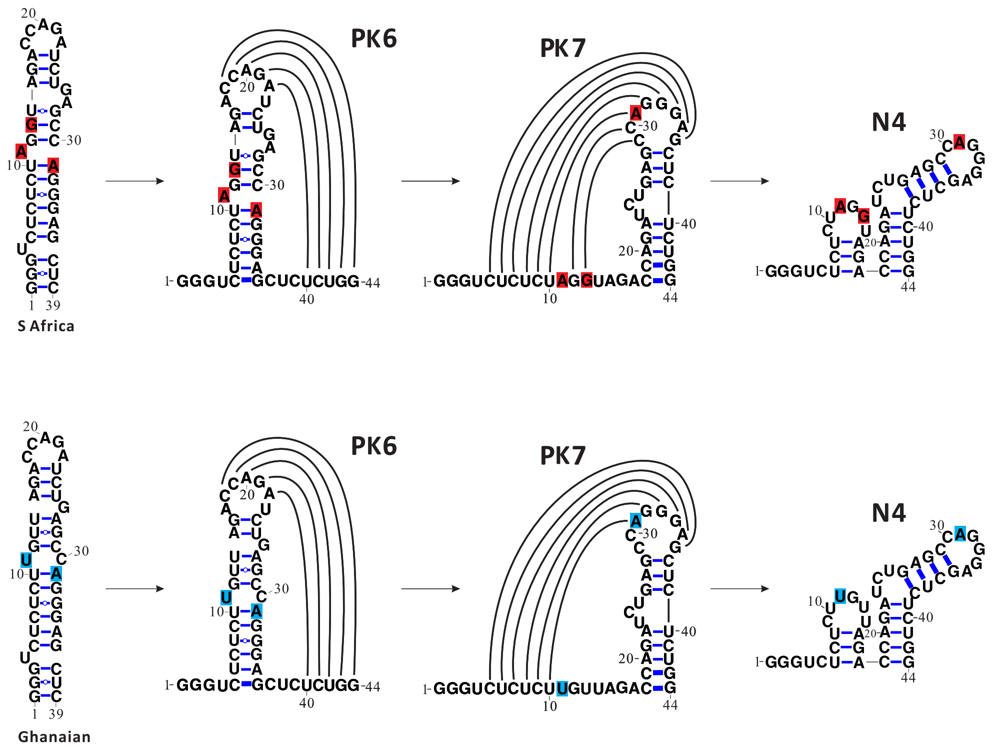

Figure S5: Pseudoknot assisted transition from misfolded structure P4 to native pathway N4 for the two mutants.

References

[1] Turner, D. H.; Mathews, D. H. NNDB: the nearest neighbor parameter database for predicting stability of nucleic acid secondary structure. *Nucleic Acids Res.* **2010**, *38*, D280-D282.

[2] Metropolis, N.; Rosenbluth, A. W.; Rosenbluth, M. N.; Teller, A. H.; Teller, E. Equation of state calculations by fast comuting machines. *J. Chem. Phys.* **1953**, *21*, 1087-1092.

[3] Xu, X.; Yu, T.; Chen, S-J. Understanding the kinetic mechanism of RNA single base pair formation. Proceedings of the National Academy of Sciences. *Proceedings of the National Academy of Sciences* **2016**, *113*, 116-21.

[4] Xu, X.; Chen, S-J. Kinetic Mechanism of Conformational Switch between Bistable RNA Hairpins. *J. Am. Chem. Soc.* **2012**, *134(30)*, pp.12499-12507.

[5] Xu, X.; Zhao, P.; Chen, S-J. Vfold: a web server for RNA structure and folding thermodynamics prediction. *PLoS ONE* **2014**, *9*, e107504.

[6] Faber, M.; Klumpp, S. Kinetic Monte Carlo approach to RNA folding dynamics using structure-based models. *Phys. Rev. E* **2013**, *88*, 052701.

[7] Lutz, B.; Faber, M.; Verma, A.; Klumpp, S.; Schug, A. Differences between cotranscriptional and free riboswitch folding. *Nucleic Acids Res.* **2014**, *42*, 2687-2696.
